# Patient's site of first access to health system influences length of delay for tuberculosis treatment in Tajikistan

**DOI:** 10.1186/1472-6963-10-10

**Published:** 2010-01-10

**Authors:** Raffael Ayé, Kaspar Wyss, Hanifa Abdualimova, Sadullo Saidaliev

**Affiliations:** 1 Swiss Tropical Institute, Swiss Centre for International Health, Socinstr. 57, 4002 Basel, Switzerland; 2 Project Sino, Rudaki prospekt proyezd 5, dom 1, Dushanbe, Tajikistan; 3 Republican Centre for Tuberculosis Control, Bukhoro street 53, Dushanbe, Tajikistan

## Abstract

**Background:**

Tajikistan has the highest incidence rate of tuberculosis (TB) in Central Asia. Its health system still bears many features from Soviet times and is under-funded. Affordability is a major barrier to health care. Little is known about health care seeking of TB patients in post-Soviet countries and their delay until the start of TB therapy. The low estimated case detection rate in Tajikistan suggests major problems with access to care and consequently long delays are likely.

**Methods:**

The study investigated extent and determinants of patient and health system delays for TB. A questionnaire was administered to a cohort of TB patients in twelve study districts representing a wide range of conditions found in Tajikistan. Common patterns of health care seeking were analysed. Cox proportional hazards models using eight predictor variables, including characteristics of health services delivery, were built to identify determinants of patient and health system delays.

**Results:**

Two-hundred-and-four TB patients were interviewed. A common pattern in treatment-seeking was visiting a specialised TB facility at some stage. Typical delays until start of TB therapy were moderate and did not confirm the expectation of long delays. Median patient, health system and total delays to TB treatment were 21.5, 16 and 52 days, respectively. None of the investigated predictors was significantly associated with patient delay. The type of facility, where patients made their first contact with the health system, was the main determinant of health system delay (p < 0.00005). We show for the first time that patients who had fallen ill and first presented to health care in Russia had the longest delays. Those who first presented to peripheral primary care facilities also had relatively long delays.

**Conclusions:**

While overall delays were moderate, further improvement is needed for different subgroups. An international referral system between Russia and Tajikistan to reduce delays of Tajik migrants who develop active TB in Russia is urgently needed and would benefit both countries. Within Tajikistan, diagnostic pathways for patients in the periphery should be shortened. To achieve this, strengthening of sputum smear examination possibly including collection of sputa at peripheral primary care facilities may be needed.

## Background

The tuberculosis (TB) incidence rate in Tajikistan is estimated at 231 cases per 100'000 population in 2007[1]. The case detection rate ranges from 32% for sputum smear positive cases to 39% for all cases. While the incidence estimate from the national TB control program is slightly lower (160 - 180 cases per 100'000), it is clear that the TB epidemic is ravaging. Integration of the vertical TB program rooted in the Soviet tradition with primary care is not yet complete. Historically, TB diagnosis and treatment was the exclusive task of specialised TB facilities and providers. The DOTS strategy was introduced in two pilot districts in 2002 and by the end of 2007 has reached 100% coverage of Tajikistan. Under DOTS, primary care providers also have the task of diagnosing and treating TB. Despite insufficient funding, the health system has been latching onto a dense network of facilities with different levels of specialisation inherited from Soviet times (table 1). Real government expenditure for health was less than US$2 per capita in Tajikistan in 2003[2]. Health care workers are poorly paid and informal payments are common [2,3]. Out-of-pocket payments are an important barrier to access health care services[4]. The policy in Tajikistan is to provide anti-TB chemotherapy free of charge. Currently, health reform is ongoing in order to strengthen primary care under the name of Family Medicine[3].

**Table 1:** Characteristics of facilities in the Tajik health system§

Facility type	Specialisation of health worker†	Hospital beds	Description
Medical House	Nurse	No	Facility serving villages without a Rural Health Centre. Together with the latter forms the peripheral primary care. Basic services from a nurse or a trained health worker.
Rural Health Centre	Generalist/family doctor	No°	Facility serving mid-sized and larger villages. Stands at the centre of Family Medicine and thus primary care. One or more family doctors and a number of nurses.
Polyclinic	Specialist doctor	No	Facility found in district centres and larger towns. Specialist doctors provide ambulatory services.
Central District Hospital	Specialist doctor	Yes	Found in district centres. Specialist doctors provide in- and out-patient services. Usually contains an ambulatory functioning like a Rural Health Centre.
District TB facility (including TB hospitals)	TB specialist	Partially	Usually in the district centre. Often more than one in large districts. Part of the historical vertical structure providing care for TB exclusively.
Republican Hospital	Specialist doctor	Yes	Tertiary referral centres providing the most specialised services available.
Private clinic	Varying	Varying	Exist only in large towns, mainly in the capital.

§This list is by no means complete, but contains the facilities of relevance to this study.

†This column shows the highest professional specialisation that is usually found at facilities of the respective type.

°Some Rural Health Centres are historically derived from rural hospitals with beds and in a transitional period may have hospital beds.

It is estimated that up to a fifth of the population of Tajikistan is working abroad, mainly in Russia, where Tajik migrant workers are very vulnerable[5]. Albeit labour migration has recently decreased due to the current economic crisis and the loss of job opportunities in Russia, remittances from labour migration are likely to remain an important component of the Tajik economy.

TB control in low-income countries relies primarily on treatment of active cases. A dose-response relationship has been found between the delay to TB treatment and the transmission to household members[6]. Consequently, prompt start of treatment is of utmost importance. Passive case finding requires that patients present to a health care facility and that health providers take appropriate measures. Repeated referrals that do not result in a diagnosis and effective treatment can frustrate patients, leading to delayed or missed diagnoses. Storla et al[7] reviewed studies on delay to tuberculosis treatment-defined as the period from onset of symptoms until the start of treatment. They found that many factors can influence delay and that the same factor could lead to shorter or longer delays in different settings. The type of the first health care provider chosen often influenced delay with findings relatively consistent among studies: patients who first visited a traditional healer, private provider, or a low-level government health facility had longer delays [8-16]. Poverty and rural residence were almost invariably associated with longer delays[7]. Factors that often led to longer but sometimes also to shorter delays were female sex and old age[7]. Despite the specific traditions of TB control in the former Soviet Union (FSU), no studies on the relationship between health services delivery and delay in a post-Soviet country were found in the scientific literature. The only study on delay from the FSU concentrated on stigma and patient delay in Russia[17]. Investigating delay to TB treatment in the FSU may contribute to the understanding of the devastating TB epidemic in this region.

The objective of the present study was to describe common health care seeking behaviours of new pulmonary TB patients in Tajikistan and to identify determinants of delay based on the following explanatory variables: sex, age, rural versus urban residence, district, durable assets, labour migration to Russia, belief in curability of TB, use of self-treatment, sputum smear result, and the type of facility first visited.

## Methods

### Study population

**Figure 1:**
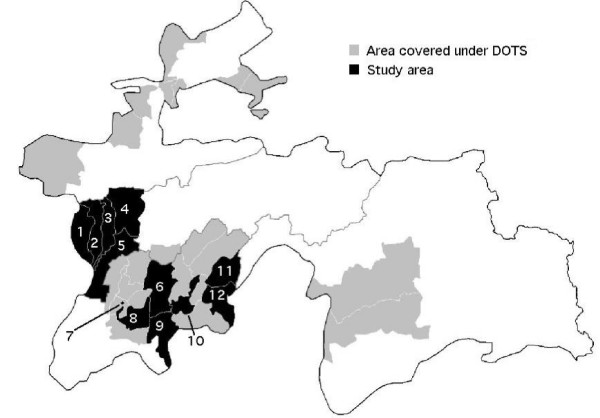
**Location of study districts among all districts with a DOTS program in July 2006 in Tajikistan**. District codes: 1 = Tursunzoda, 2 = Shahrinaw, 3 = Hissor, 4 = Varzob, 5 = Rudaki, 6 = Danghara, 7 = Qurghonteppa, 8 = Vakhsh, 9 = Farkhor, 10 = Vose, 11 = Muminobod, 12 = Shurobod.

As DOTS expansion was under way in Tajikistan and in order to produce meaningful evidence for the future, we included only districts with an established DOTS program (started in July 2006 at the latest) in the study. Two regions were excluded because they were accessible only by air at the time of the study, Badakhshan and Sughd. Among the 24 remaining districts ('rayons'), twelve were purposefully selected to represent urban, rural, lowland and mountainous settings (figure 1).

### Questionnaire and Interviews

All patients who fulfilled the inclusion criteria and who were registered in the twelve study districts were recruited into the study. Our inclusion criteria were: enrolled for TB treatment in the period 1 December 2006 to 31 March 2007, new pulmonary tuberculosis case (sputum smear positive or negative) and at least 15 years old. Data for this study were collected alongside a study on household costs of an episode of TB[18]. The latter study included two interviews with each patient. For the present study, only data from the first interview were analysed. Patients were visited in hospital or at their homes during the intensive phase of treatment, written informed consent was obtained and a trained interviewer administered a questionnaire. The questionnaire included detailed questions about the onset of symptoms, health care seeking behaviour (including traditional healers and self-treatment), the timing of health care seeking steps, diagnostic investigations that were conducted and household assets. Onset of symptoms was self-reported and concerned any symptoms likely to be due to TB, most importantly cough. Patient delay was defined as the period from onset of symptoms until the first visit to a formal health care provider. Formal health care providers included all the facilities listed in table 1, but not traditional healers nor pharmacies, as they are not allowed to diagnose and treat patients in the Tajik health system. Health system delay was defined as the time from the first visit to a formal health care provider to the start of treatment.

Interviewers were trained during a fortnight before conducting interviews on their own and were supervised at least twice per month through joint interviews with the main researcher. Specific emphasis was given to support the patients in their recall efforts, including the use of a calendar of locally important events. Data were entered in FileMaker (version 8.0v1, FileMaker Inc, USA, 1984-2005).

### Data Analysis

All analyses were conducted in Stata IC/10.1 for Macintosh (Stata Corporation, USA, 1985-2008). We carried out a principal component analysis on 15 household asset variables and three housing characteristics variables to construct a wealth index using a well established and validated method [19,20]. The wealth index was used to assign patients to quintiles of socio-economic status (SES).

For each patient, we listed every health care seeking step and identified common patterns in the history of health care seeking up to the start of anti-TB chemotherapy. Particular attention was given to the timing and the place of the sputum smear examination.

In order to investigate delay, we built Cox proportional hazards models with delay as time-to-event. Two separate models were built for patient and system delay, because predictor variables are likely to differ. Sex, age (categorised into four groups), residence, SES quintiles, district, and labour migration were included in both models. Belief in curability of TB and use of self-treatment were included only in the model on patient delay. Sputum smear status and the type of facility visited in first instance were included only in the model for system delay. Knowledge of symptoms was not included, as the encounters with health care professionals could change this variable between the times when it acts (before the first visit to a formal provider) and when it is measured (during treatment). We selected the best model by subsequently excluding factors with p > 0.2 in the likelihood-ratio test from the full model, until no more factors could be excluded. The respective univariate models were also built and the hazards ratios of factors were compared between univariate and multivariate models in order to investigate potential confounding.

The present study has received approval from the Ministry of Health of the Republic of Tajikistan in a memorandum of understanding signed 18^th ^October 2006.

## Results

### Study population and health care seeking

The study identified 282 eligible patients, of whom 78 dropped out (table 2) and 204 were interviewed (table 3). The patients who dropped out were virtually identical to included patients in terms of the proportions of smear-positive (60%in both cases) and male patients (54% versus 56%), but differed somewhat in their age-distribution and in terms of residence. The proportion of patients who were at least 45 years old was 36% among dropouts versus 16% amongst the study sample. The proportion of rural patients was 79% versus 90%.

**Table 2:** Reasons for dropout

Reason	Number of cases (% of drop-outs)
Wrong or insufficient address	18 (23)
Not found at home or in hospital when visited	17 (22)
Died	5 (6)
Initial defaulter	3 (4)
Already in continuation phase when visited	25 (32)
Did not consent	10 (13)
**Total**	**78 (100)**

**Table 3:** Demographic characteristics of study sample

	Women	Men	Total
**Profession**	**n (%)**	**n (%)**	**n (%)**
Unemployed	9 (10)	20 (18)	29 (14)
Student	4 (4)	12 (11)	16 (8)
Housewife	52 (58)	0 (0)	52 (25)
Retiree	5 (6)	2 (2)	7 (3)
Employee in industry	1 (1)	7 (6)	8 (4)
Employee in private service	0 (0)	2 (2)	2 (1)
Owner of business	0 (0)	0 (0)	0 (0)
Police	0 (0)	3 (3)	3 (1)
Health Care Worker	1 (1)	1 (1)	2 (1)
Civil service (other than police and health)	3 (3)	5 (4)	8 (4)
Petty Trader	3 (3)	19 (17)	22 (11)
Labour Migration to Russia	1 (1)	30 (26)	30 (15)
Farmer	11 (12)	13 (11)	24 (12)
**Total**	**90 (100)**	**114 (100)**	**204 (100)**
			
**Age**			
15-24 y	34 (38)	44 (39)	78 (38)
25-34 y	23 (26)	40 (35)	63 (31)
35-44 y	16 (18)	14 (12)	30 (15)
45 y and older (max 72 y)	17 (19)	16 (14)	33 (16)
**Total**	**90 (100)**	**114 (100)**	**204 (100)**
			
**Residence**			
Rural	79 (88)	105 (92)	184 (90)
Semi- or Peri-urban	3 (3)	8 (7)	11 (5)
Urban	8 (9)	1 (1)	9 (4)
**Total**	**90 (100)**	**114 (100)**	**204 (100)**

Self-treatment was used by 64 patients (31%) before the start of anti-TB chemotherapy. A large variety of self-treatments were used, ranging from honey and different herbs over meat and fat of boar or dog to opium and allopathic medicines. Self-treatment was used both before and after the first visit to a formal health care provider. Only one patient (0.5%) reported having visited a traditional healer before the first visit to a formal health care provider and four more patients did so after such visit.

The first formal health care facility approached was a Rural Health Centre for 36 patients (18%), a central district hospital for 39 patients (19%) and the district TB facility for 41 (20%) patients (table 4). None of the study subjects visited a private clinic. Each facility that was visited was visited approximately twice on average (table 4, right-hand column). Patients made 4.8 (± s.e. 0.17) visits to formal health care facilities on average before anti-TB chemotherapy was started. It is noteworthy that the district TB facilities were visited at some stage before initiation of anti-TB chemotherapy by a large majority (86%) of patients. Of the 28 patients who had not visited their district TB facility, 15 lived in Danghara district and all 15 had visited the Central District Hospital. In Danghara there was no separate TB facility as it had already been integrated in the Central District Hospital.

**Table 4:** Health care facility visited by TB patients

	#patients visiting facility in first instance	mean #visits (all patients)	#visits among patients who visited§
Medical house	22 (10.8%)	0.23	1.88
Rural Health Centre	36 (17.7%)	0.45	2.04
Polyclinic	17 (8.3%)	0.28	2.19
Private clinic	0 (0.0%)	0.00	0.00
Central District Hospital	39 (19.1%)	0.83	1.77
Republican Hospital	15 (7.4%)	0.53	1.96
District TB facility	41 (20.1%)	1.86	2.16
Health facility in Russia	20 (9.8%)	0.25	2.60
Other facility†	14 (6.9%)	0.39	1.65
**Sum**	**204 (100%)**	**4.83**	**N/A**

§Mean number of visits among those patients who visited the respective facility.

†Including a health care facility reserved to staff of a large industrial company and Ministry of Defence facilities.

### Delay

The median patient delay was 21.5 days (range: 0 to 410 days; mean: 45.7 days). Thirty-six patients were excluded from the analysis of patient delay, because they reported either no symptoms, a chronic pulmonary condition, or because they were unable to assign at least an approximate date to both the onset of cough and the first visit to a health care provider. The median reported system delay was 16.0 days (0 to 339; 42.2). One patient was excluded from the analysis of system delay because he could not assign a date to the first visit to a formal provider. The median total delay from onset of cough until start of anti-TB chemotherapy was 53 days (2 to 542; 81.8). Note that mean delays are not additive because of the 36 patients excluded from analysis of patient delay, but not system delay.

### Determinants of Delay

None of the factors included in the model on patient delay proved significant. Sex (hazards ratio women to men 0.745, p = 0.103) and SES quintile (p = 0.132) were retained in the final model (table 5). The final model on system delay included the factors 'first facility' and 'district' (table 6). The proportional hazards assumption was not violated (based on Schoenfeld residuals, p = 0.91). There was a striking relationship between first visiting a district TB specialist and system delay: the median was six days versus 23 days for all other facilities combined. Also, visiting first a Central District Hospital was associated with a short system delay. Visiting first a Medical House, Rural Health Centre, Polyclinic or a facility in Russia was associated with longer system delays. Patients who had first visited a health facility in Russia had the longest system delays of all. It was found that most patients were advised at the health facility in Russia to travel to Tajikistan for treatment - often already with a diagnosis or at least suspicion of TB. Several patients reported that the Russian health system made no treatment available to them.

**Table 5:** Results of the multivariate Cox proportional hazards model on patient delay (n = 168)

Factor	Hazards Ratio§	Lb95%CI†	Ub95%CI°	p-value
Male sex*	1.000	NA/	NA/	NA/
Female sex	0.745	0.523	1.061	0.103
				
**SES quintile**	**NA**	**NA**	**NA**	**0.132¶**
SES quintile 1*	1.000	NA	NA	NA
SES quintile 2	0.661	0.409	1.067	0.090
SES quintile 3	1.045	0.645	1.694	0.859
SES quintile 4	0.614	0.365	1.032	0.065
SES quintile 5	0.712	0.432	1.174	0.183

§A hazards ratio >1 means a shorter delay, a hazards ratio <1 points to a longer delay relative to the comparison group.

†Lb95%CI = Lower boundary of 95%-confidence interval

°Ub95%CI = Upper boundary of 95%-confidence interval

*Comparison group

¶Based on log-likelihood ratio test

**Table 6:** Results of the multivariate Cox proportional hazards model on health system delay (n = 203)

Factor	Hazards Ratio§	Lb95%CI†	Ub95%CI°	p-value
**District**	**NA**	**NA**	**NA**	**0.0191¶**
Danghara*	1.000	NA	NA	N/A
Farkhor	0.917	0.490	1.716	0.786
Hissor	1.243	0.639	2.417	0.522
Muminobod	0.412	0.208	0.817	0.011
Qurghonteppa	1.502	0.623	3.623	0.365
Rudaki	1.516	0.837	2.744	0.170
Shahrinaw	1.293	0.291	5.738	0.735
Shurobod	1.335	0.600	2.968	0.479
Tursunzoda	0.966	0.509	1.835	0.916
Vakhsh	1.462	0.769	2.781	0.247
Varzob	0.953	0.334	2.720	0.928
Vose	0.879	0.488	1.584	0.669
				
**First facility**	**NA**	**NA**	**NA**	**< 0.00005¶**
District TB facility*	1.000	NA	NA	NA
Medical House	0.325	0.183	0.580	< 0.0005
Rural Health Centre	0.418	0.255	0.688	0.001
Polyclinic	0.409	0.216	0.773	0.006
Central District Hospital	0.704	0.435	1.136	0.151
Republican Hospital	0.646	0.341	1.226	0.181
Health facility in Russia	0.212	0.117	0.383	< 0.0005
Other facility‡	0.365	0.185	0.721	0.004

§A hazards ratio >1 means a shorter delay, a hazards ratio <1 points to a longer delay relative to the comparison group.

†Lb95%CI = Lower boundary of 95%-confidence interval

°Ub95%CI = Upper boundary of 95%-confidence interval

¶Based on log-likelihood ratio test

*Comparison group

‡Including health care facilities reserved to staff of a large industrial company or the Ministry of Defence, respectively.

In order to check for potential confounding, the 16 univariate models that are relevant to the above two multivariate models were also computed. The univariate models on patient delay showed consistent results with all hazards ratios in the same direction and in a similar range (table 7). Among the univariate models on health system delay (table 8), the factor 'labour migration' showed a strong association in the univariate model (hazards ratio 0.563, p = 0.006) but showed no significant relationship (at the p = 0.2 level) in the multivariate model and was consequently excluded.

**Table 7:** Results of univariate Cox proportional hazards models on patient delay (n = 168)

Factor	Hazards Ratio§	Lb95%CI†	Ub95%CI°	p-value
Male sex*	1.000	NA/	NA/	NA/
Female sex	0.834	0.613	1.136	0.250
				
**Age**	**NA/**	**NA/**	**NA/**	**0.562**
15-24 y*	1.000	NA/	NA/	NA/
25-34 y	0.870	0.598	1.265	0.466
35-44 y	0.827	0.527	1.299	0.409
45 y and older	0.728	0.458	1.157	0.179
				
**Residence**	**NA/**	**NA/**	**NA/**	**0.831**
Semi- or peri-urban*	1.000	NA/	NA/	NA/
Rural	0.903	0.459	1.777	0.767
Urban	0.751	0.288	1.961	0.559
				
**SES quintile**	**NA/**	**NA/**	**NA/**	**0.221**
SES quintile 1*	1.000	NA/	NA/	NA/
SES quintile 2	0.669	0.414	1.080	0.100
SES quintile 3	1.116	0.693	1.798	0.651
SES quintile 4	0.730	0.454	1.175	0.195
SES quintile 5	0.828	0.520	1.319	0.428
				
**District**	**NA/**	**NA/**	**NA/**	**0.755**
Danghara*	1.000	NA/	NA/	NA/
Farkhor	0.898	0.468	1.724	0.747
Hissor	0.639	0.315	1.297	0.215
Muminobod	1.023	0.496	2.111	0.95
Qurghonteppa	0.833	0.334	2.079	0.696
Rudaki	0.717	0.393	1.307	0.277
Shahrinaw	4.935	0.643	37.845	0.125
Shurobod	1.507	0.636	3.573	0.351
Tursunzoda	0.859	0.449	1.645	0.647
Vakhsh	0.751	0.390	1.446	0.392
Varzob	0.544	0.184	1.607	0.271
Vose	0.873	0.492	1.549	0.642
				
No migration*	1.000	NA/	NA/	NA/
Labour migration	0.889	0.565	1.400	0.612
				
Lack of belief in curability*	1.000	NA/	NA/	NA/
Belief in curability	1.121	0.827	1.518	0.462
				
No self-treatment*	1.000	NA/	NA/	NA/
Self-treatment	1.011	0.727	1.406	0.949

§A hazards ratio >1 means a shorter delay, a hazards ratio <1 points to a longer delay relative to the comparison group.

†Lb95%CI = Lower boundary of 95%-confidence interval

°Ub95%CI = Upper boundary of 95%-confidence interval

*Comparison group

**Table 8:** Results of univariate Cox proportional hazards models on health system delay (n = 203)

Variable	Hazards Ratio§	Lb95%CI†	Ub95%CI°	p-value
Male sex*	1.000	NA/	NA/	NA/
Female sex	1.201	0.908	1.589	0.200
				
**Age**	**NA/**	**NA/**	**NA/**	**0.270**
15-24 y*	1.00	NA/	NA/	NA/
25-34 y	0.777	0.556	1.087	0.141
35-44 y	1.126	0.736	1.721	0.584
45 y and older	0.810	0.535	1.226	0.319
				
**Residence**	**NA/**	**NA/**	**NA/**	**0.682**
Semi- or periurban*	1.000	NA/	NA/	NA/
Rural	1.066	0.579	1.965	0.836
Urban	1.441	0.594	3.494	0.419
				
**SES quintile**	**NA/**	**NA/**	**NA/**	**0.442**
SES quintile 1*	1.000	NA/	NA/	NA/
SES quintile 2	0.946	0.608	1.471	0.804
SES quintile 3	1.004	0.646	1.558	0.987
SES quintile 4	0.781	0.501	1.219	0.277
SES quintile 5	1.205	0.779	1.865	0.402
				
**District**	**NA/**	**NA/**	**NA/**	**0.368**
Danghara*	1.000	NA/	NA/	NA/
Farkhor	1.043	0.564	1.930	0.892
Hissor	1.189	0.631	2.240	0.593
Muminobod	0.680	0.362	1.279	0.232
Qurghonteppa	1.725	0.734	4.052	0.211
Rudaki	1.553	0.895	2.694	0.118
Shahrinaw	0.857	0.201	3.653	0.835
Shurobod	1.418	0.650	3.092	0.380
Tursunzoda	1.284	0.696	2.368	0.423
Vakhsh	1.526	0.822	2.831	0.180
Varzob	1.311	0.495	3.472	0.586
Vose	1.180	0.684	2.038	0.551
				
No migration*	1.000	NA/	NA/	NA/
Labour migration	0.563	0.375	0.846	0.006
				
Sputum smear negative*	1.000	NA/	NA/	NA/
Sputum smear positive	1.153	0.870	1.530	0.322
				
**First facility**	**NA/**	**NA/**	**NA/**	**0.0003**
TB facility*	1.000	NA/	NA/	NA/
Medical House	0.425	0.249	0.724	0.002
Family Doctor	0.442	0.281	0.697	< 0.0005
Central District Hospital	0.768	0.492	1.196	0.243
Policlinic	0.562	0.317	0.996	0.049
Republican Hospital	0.613	0.338	1.111	0.107
Russia	0.302	0.175	0.524	< 0.0005
Other‡	0.478	0.259	0.882	0.018

§A hazards ratio >1 means a shorter delay, a hazards ratio <1 points to a longer delay relative to the comparison group.

†Lb95%CI = Lower boundary of 95%-confidence interval

°Ub95%CI = Upper boundary of 95%-confidence interval

*Comparison group

‡Including health care facilities reserved to staff of a large industrial company or the Ministry of Defence, respectively.

## Discussion

This study investigated the influence of health services delivery rooted in the Soviet tradition of vertical TB control programs on delay to TB treatment. To our knowledge there have been no similar studies previously. We found generally moderate delays in our study sample. Much longer delays for certain subgroups of patients highlight the need for improvements in the organisation of health services delivery. These improvements include the implementation of an international referral system between Russia and Tajikistan.

### Limitations

As in any study on self-reported delay, there is potential recall and reporting bias: patients might not remember exactly (or even realise) when the first symptoms started, thus they might report delay incorrectly. We have tried to minimise recall and reporting bias by supporting the interviewees in their recall work-among others using a calendar of local events-and by carefully training interviewers to be non-judgmental in the inquiries about delay.

Residence broken up into the three levels rural, semi- or peri-urban and urban, is a very approximate measure of geographical accessibility. However, during the pilot study patients could not give estimates of the distance in kilometres from their place of residence to the nearest health care facility. We therefore included residence in our models on patient and system delays-a factor which may include other information in addition to geographical accessibility. The small number of patients from urban and peri-urban areas made it difficult to detect statistical differences by residence. Medical Houses and Rural Health Centres exist in very remote places and it is likely that remoteness contributed to the long delays found in patients who first visited one of these two facility types. Residence, which was included in the analysis, could only partly account for this because many district centres (where a TB facility and a hospital are situated) were also classified as rural.

The sample of patients for our study did not span a full year. Consequently, we cannot exclude that delays differ during other seasons.

It is possible that some patients went by without ever being registered in the TB registry-for instance if patients relied on self-treatment or informal providers exclusively. The present study might consequently underestimate some aspects of difficulty in access and delay to TB treatment. This could affect our secondary finding that this study did not find evidence of the very strong barriers to access that might explain the low case detection rate in Tajikistan. However, such underestimation of difficulties in access is most likely for Tajik TB patients in Russia, which would reinforce our findings if anything.

### Health care seeking

Patients in our study group made on average 4.8 visits to a formal health care provider up to anti-TB chemotherapy. This is more than the two to three visits that the diagnostic pathway requires, namely two visits to conduct sputum smear examination (usually at the district TB facility) and possibly one additional visit at the peripheral level, from where patients are referred. However, it is less than what was found for example in urban Zambia, where TB patients made 6.7 health care visits on average[21].

In this study there was a lot of variability in the facility type, where the first contact with the health system took place: eight of the nine facility types accounted for a considerable proportion (6.9% or more) of first contacts each. This is a broader variety than found by Mfinanga et al[22] in Tanzania. Strikingly, almost all patients visited the district TB facility at some time during health care seeking. Especially primary care providers tend to refer patients to TB specialists rather than diagnosing TB themselves. At the district TB facility, usually both chest x-ray (CXR) and sputum smear examination were conducted. Similar to a study in the Philippines[23], we found that almost all patients had had a CXR - despite national and international guidelines not requiring CXR for sputum smear positive patients.

Eighty-six percent of patients reported a visit to the TB specialist. Fifteen patients who did not report such visit came from Danghara and had visited the Central District Hospital there, which includes a TB ward. Hence, the proportion of patients who have been seen by a TB specialist is likely even higher than 86%.

The patients in our study sample reported very limited use of informal providers. Social desirability bias could be suspected. However, interviewees freely reported their experiences in other areas where social desirability bias could be suspected such as in the area of informal payments. We therefore believe that social desirability bias is not the reason for the low use of informal providers reported, but that TB patients in Tajikistan really preferred formal health care providers.

### Delay

Given the heavily resource-constrained setting, the total delay found in this study is moderate. It is longer than those reported in the Philippines and in China (median delay of one month in both cases[15,23]), but much shorter than found in studies in high-incidence settings, where the median delay ranged from 80 to 120 days[7,11-13,24]. This result is encouraging and to a certain extent surprising. The estimated case detection rate according to data of the World Health Organisation is very low[1] and suggests that TB patients face major barriers to care and many of them remain undetected. The reasonable median delay found in this study does not necessarily support this view.

### Determinants of delay

District and type of first facility visited were significant determinants of health system delay. Muminobod is a mountainous district and this may be one of the reasons why treatment delays were longest there (cf. figure 1). On the other hand, the similarly mountainous neighbouring district of Shurobod was among the districts with relatively short delays. Differences between districts may account for a multitude of factors including differences in socio-economic conditions, accessibility, or practices in the health centres in the district. The type of the facility where the first formal health care contact took place was the strongest predictor of system delay. Visiting first a tertiary hospital or TB specialist is associated with shorter delays. This is consistent with findings from other settings, although there is considerable variation[11-16,25]. Making the first visit to a Central District Hospital was also significantly associated with shorter delay. While a history of immigration has been shown to influence delay in western countries [25,26], this study shows for the first time that developing active TB while being a temporary migrant worker abroad leads to long system delays: patients who first visited a facility in Russia had the longest delays of all. This is not surprising given that these patients fell ill in Russia and had to travel back to Tajikistan to start anti-TB chemotherapy. About 10% of all TB patients receiving treatment in the study area had developed active TB in Russia. This may also be relevant for citizens of several other Central Asian and Caucasian countries who migrate to Russia to find work. The moderate delays of many patients are encouraging, but improvements are needed for patients first presenting to Medical Houses, Rural Health Centres and especially facilities in Russia.

The consistent results of the univariate analyses compared to the multivariate analysis support the view that these results are not due to confounding. The factor 'labour migration' is an exception in the sense that it shows a significant impact on health system delay in the univariate but not in the multivariate analysis. There is strong correlation between 'labour migration' and having visited a facility in Russia. This does, however, not affect our conclusions.

In order to reduce delays for Tajiks who develop active TB while working in Russia, an international referral system should be developed. The Russian TB program and international organisations involved in TB control in Russia should investigate the options of foreigners who develop TB while in Russia. If the situation is as described by the patients in our study sample, there are disincentives to early presentation to the Russian health system, because the patient cannot expect treatment. Rather, presenting to the Russian health system will mean that patients will have to spend money to return to their home country. Such a disincentive is likely to lead to delayed diagnosis and consequently to extended periods of infectiousness in the community.

As mentioned above, most providers other than TB specialists are reluctant of diagnosing TB and would rather refer the patient. This is related to the vertical TB control program inherited from the Soviet Union and the resulting notion that TB patients should be diagnosed and treated outside the general health services. The large difference in the hazards ratios from the peripheral facilities to the TB facility point at a need to shorten referral pathways and referral times for TB patients from the periphery. This needs to be achieved without jeopardizing the short delays that patients experience at the TB facilities. A good possibility would be the diagnosis of TB in the peripheral facilities without referral. At least for part of the districts, a sputum collection plan exists, whereby peripheral facilities are required to collect three sputa from a TB suspect and then organise transport to the district TB facility, where microscopic examination is conducted. Due to the Soviet tradition of over-reliance on CXR and to doubts about accuracy of sputum smear microscopy, sputum collection at the periphery hardly ever takes place.

Lönnroth et al[27] have pointed out that the perceived financial consequences of seeking health care affect the decision of whether and when to initiate health care seeking. Community members, TB patients and health care providers in four districts of Tajikistan identified financial costs of health care seeking as the main obstacle to obtaining a diagnosis and treatment[28]. Consequently, a better understanding of illness-related costs and resulting mitigation strategies may also contribute to shorter delays.

## Conclusions

Before being diagnosed, TB patients visit a broad range of different health facilities. While the median total delay until start of treatment is moderate for most patients, improvements are needed for certain subgroups. Those patients who develop TB while working in Russia have the longest health system delays. Those who first present to peripheral primary care facilities or polyclinics have long system delays, too. Improvements for both subgroups are possible through better organisation of health care delivery. An international referral system for migrant workers developing active TB in Russia is urgently needed and is in the interest of both countries. Primary care providers should be enabled and encouraged to diagnose TB and start patients on treatment. Strengthening of sputum smear examination and collection of specimens at primary care facilities should be considered.

## Competing interests

The authors declare that they have no competing interests.

## Authors' contributions

RA led the study from planning to writing up. KW contributed to designing the study and to writing the manuscript. HA and SS participated in design of the study and data collection. All authors read and approved the final manuscript.

## Pre-publication history

The pre-publication history for this paper can be accessed here:

http://www.biomedcentral.com/1472-6963/10/10/prepub
